# Effects of thermal processing on the nutritional and functional properties of defatted conophor nut (*Tetracarpidium conophorum*) flour and protein isolates

**DOI:** 10.1002/fsn3.508

**Published:** 2017-08-27

**Authors:** David O. Iyenagbe, Sunday A. Malomo, Atinuke O. Idowu, Adebanjo A. Badejo, Tayo N. Fagbemi

**Affiliations:** ^1^ Department of Food Science and Technology Federal University of Technology Akure Nigeria; ^2^ Department of Food Science and Technology Mountain Top University Ibafo Ogun State Nigeria; ^3^ Department of Agricultural Biotechnology Assam Agricultural University Jorhat Assam India

**Keywords:** amino acid composition, conophor nut, functional properties, heat processing, protein isolates

## Abstract

Conophor nut (*Tetracarpidium conophorum*) was processed using different heat treatments to explore its full potential as food ingredients. The raw, boiled, and toasted nuts were defatted and the proteins isolated by alkaline solubilization and isoelectric precipitation. The variously processed nuts were analyzed for the proximate and amino acid compositions, and functional properties. The protein contents of the isolate ranges between 86.86 g/100g and 87.74 g/100 g, about 1.5‐fold higher than those of the defatted flour samples. The essential amino acids of the isolates ranged between 40.57%–41.55%. Glutamic acid, aspartic acid, and arginine were the most predominant amino acids, while methionine and lysine were the first and second limiting amino acids, respectively. The protein efficiency ratio, biological values as well as the functional properties of the proteins were improved with processing. These properties may enhance the potential use of conophor nut protein isolates as high‐quality protein ingredient in food systems.

## 1 INTRODUCTION

Animal protein is still far more expensive than their plant counterpart for most dwellers of the developing countries, and for years this has aroused the rigorous researches on underutilized legumes and oilseeds that can be used as protein source in food ingredients and functional foods formulation (Chavan, McKenzie, & Shahidi, 2001; Sreerama, Neelam, Sashikala, & Pratape, 2010). The nutritional and functional properties of protein isolate from legumes and oilseeds has increased their rate of application in meats and sausages, baked and extruded products, salad dressings and frozen desserts (Asgar, Fazilah, Huda, Bhat, & Karim, 2010; Day, 2013).

Conophor nut (*Tetracarpidium conophorum*), commonly called African walnut, is one of the greatly underutilized oilseeds belonging to the family *Euphorbiaceae*. The monoecious and high‐climbing plant produce seeds which become dark brown with hard testa when mature. Its economic importance lies in the edibility of its oil‐rich seeds which are consumed in Sub‐Saharan Africa (Enujiugha, 2008). The nut is rich in minerals and high‐quality proteins (Odoemelam, 2003; Edem, Dosunmu, & Bassey, 2009). This makes it a potential plant protein that can supplement the monotonous starchy staples being consumed by many in developing countries with little or no alternatives.

The bitter after‐taste of raw conophor nut upon reaction with water has greatly limited its use in food product development. Till date, neither is there any final product application reported for the oil‐rich seed nor any use as protein supplement. Processing through the application of heat has been reported to reduce antinutritional factors and toxic contents in nuts and legumes resulting in more effective use of the seeds (Devappa & Swamylingappa, 2008; Arinola & Adesina, 2014). Toasting, aside from reducing antinutrients, improves the taste and nutrient quality of food products and lowers the moisture contents, thus increasing the shelf life. The amino acid composition and functional properties of cashew nut were reported to have improved due to processing (Fagbemi, 2009).

In recent times, the food industries employ more of the protein concentrates and isolates from plants rather than the flour protein with their naturally occurring matrices that include carbohydrates and lipids (Arogundade, Eromosele, Ademuyiwa, & Eromosele, 2009; Shevkani, Kaur, Kumar, & Singh, 2015). Soy and wheat are among the most used plant protein isolate and concentrates, however, due to the allergenicity of these plants, the search for alternative is imperative (Boye et al., 2010). The full‐fat seed, defatted flour, and protein isolate of raw conophor nut had been shown to exhibit several desirable functions that could enhance its use in the food system (Enujiugha, 2003; Ndie, Nnamani, & Oselebe, 2010; Gbadamosi, Abiose, & Aluko, 2012a). However, there is the need for improvement on these functionalities to further enhance the desirability of conophor nut as a food ingredient.

In this study, conophor nut was processed by boiling and toasting and the effect of these heat processing operations on the nutritional quality and functional characteristics of the defatted oil seeds and protein isolates were analyzed along with the amino acid composition. This is expected to provide information on the possible uses of conophor proteins as potential functional ingredients and food supplements.

## 2 MATERIALS AND METHODS

### 2.1 Sample collection and preparation

Matured, fresh conophor nuts were obtained from a market near the Federal University of Technology Akure, Nigeria. The nuts were washed to remove dirt and divided into three parts. A part was boiled at 100°C for 30 min and another portion was toasted at 120°C for 45 min, while the third part was used in the raw form. All the nuts were shelled separately and comminuted using mortar and pestle to obtain coarse flour, which were then further ground in a mill (Retsch, Germany) and kept at 4°C for further analyses.

### 2.2 Preparation of defatted conophor nut flour and protein isolates

The various samples of conophor nuts were defatted as described by Omowaye‐Taiwo, Fagbemi, Ogunbusola, and Badejo (2015) and the resultant flour stored at 4°C for further analyses. Protein isolates were prepared from the defatted flour samples using isoelectric point method as described by Gbadamosi, Abiose, and Aluko (2012b) with slight modification. Briefly, the flour:water (1:20 [w/v]) was stirred on a magnetic stirrer continually for 2 hr at room temperature at pH 10, and the slurry formed was centrifuged at 4,000 × *g* for 40 min at 4°C. The insoluble conophor nut residue was redissolved with pH adjusted as above and cold‐centrifuged again. The two supernatants were mixed together and adjusted to pH 4.5 and kept for 2 hr at room temperature and subsequently centrifuged at 4,000 x *g* for 40 min at 4°C. The precipitate was washed with distilled water, redissolved in water, neutralized to pH 7 at room temperature, dialyzed for 6 hr and then freeze‐dried.

### 2.3 Determination of chemical and amino acid compositions of the conophor products

Proximate composition of the defatted conophor nut flour and the protein isolate was determined according to the standard procedures of Association of Official Analytical Chemists (AOAC, 1990). The micro Kjeldahl nitrogen method was used in the determination of the crude protein content. A conversion factor of 6.25 was used to convert the nitrogen content to protein. The carbohydrate content was determined by difference (100 − [moisture + total ash + crude fat + crude fiber + protein]).

HPLC system was used to determine the amino acid profiles of the protein isolates after hydrolyzing for 24 hr with 6 mol/L HCl as described by Bidlingmeyer, Cohen, and Tarvin (1984). The sulfur amino acids, cysteine, and methionine, were determined after oxidizing with performic acid (Gehrke, Wall, Absheer, Kaiser, & Zumwalt, 1985) and tryptophan content was determined using spectrophotometer after alkaline hydrolysis (Landry & Delhaye, 1992).

### 2.4 Predicted biological value, protein efficiency ratio, and Amino acid score

The predicted biological value (BV) of the protein isolates was obtained using the regression equation described by Chavan et al. (2001) as shown below:

$$\text{BV}=10^{2.15}\times q_{\mathrm{Lys}}^{0.41}\times q_{\mathrm{Phe}+\mathrm{Tyr}}^{0.60}\times q_{\mathrm{Met}+\mathrm{Cys}}^{0.77}\times q_{\mathrm{Thr}}^{24}\times q_{\mathrm{Trp}}^{0.21}$$

where,

$$q=\frac{a_{i\;\mathrm{Sample}}}{a_{i\;\mathrm{Reference}}}$$

for *a*_*i*_ Sample ≤ *a*_*i*_ Reference

$$q=\frac{a_{i\;\mathrm{Sample}}}{a_{i\;\mathrm{Reference}}}$$

for *a*_*i*_ Reference ≥ *a*_*i*_Sample

The predicted protein efficiency ratio (PER) was calculated from the amino acid compositions of the various conophor nut samples using previously described equation (Alsmeyer, Cunningham, & Happich, 1974) as shown below:

PER=−0.468+0.454(Leu)−0.105(Tyr)

Egg was used as reference protein (FAO/WHO, 2007) and amino acid score was calculated based on the composition of amino acids in the sample as described by Chavan et al. (2001) and shown in the equation:

$$\text{Amino acid score}=\frac{\text{mg of amino acid per gram test protein}}{\text{mg of amino acid per gram reference protein}}\times 100$$

### 2.5 Functional properties

The water absorption capacity (WAC) and oil absorption capacity (OAC) were determined as described by Omowaye‐Taiwo et al. (2015) adapted from Rodriguez‐Ambriz, Martinez‐Ayala, Millan, and Davila‐Ortiz (2005). Briefly, sample (1.0 g) was weighed into a 15 ml pre‐weighed centrifuge tube to which 10 ml of distilled water (for WAC) or 10 ml of soybean oil (for OAC) was added stepwise with continuous stirring at room temperature for 10 min. The suspension in the tube was centrifuged at 2,500 × *g* for 20 min and the volume of supernatant measured. The WAC or OAC was calculated as the difference between the initial volume of water or oil used and the final volume of the decanted supernatant and calculated in percentages, taking into consideration the density of the oil.

The least gelation concentration (LGC) was determined according to the method described by Abbey and Ibeh (1988). The sample was weighed and mixed with 5 ml of distilled water in a test tube to obtain 2%–20% (w/v) concentrations. The test tube was heated for 1 hr in a boiling water bath followed by rapid cooling under running tap water and further cooled for 2 hr in a refrigerator at 4°C. The LGC was regarded as the least concentration at which the sample from the inverted tube did not fall or slip.

The bulk density was determined essentially as described by Omowaye‐Taiwo et al. (2015). Foaming capacity (FC) and foaming stability (FS) were carried out as described by Sze‐Tao and Sathe (2000). The sample (0.5 g) was dispersed in 50 ml of distilled water in a 100 ml graduated cylinder and the solutions homogenized at a speed of 1,600 × *g* for 5 min. The volume was recorded before and after whipping. FC was expressed as the volume (%) increase due to whipping. This was then stored for 1 hr and the foam‐volume changes in the graduated cylinder were recorded as FS. Both were calculated in percentages as shown below:

$$\text{Foaming capacity}=\frac{\text{Volume after homogenization}-\;\text{Volume before homogenization}}{\text{Volume before homogenization}}\times 100\%$$

$$\;\text{Foaming stability}=\frac{\text{The volume of foam after a set time}}{\text{Initial volume of foam}}\times 100\%$$

Emulsion capacity (EC) was determined according to the method of Chavan et al. (2001). One gram of the sample in 25 ml distilled water was homogenized at a speed of 5,000 × *g* for 1 min at 27°C. The protein solution was then mixed with 25 ml of soybean oil followed by homogenization at 10,000 × *g* for 1 min. The emulsion volume was then used in calculating the EC as shown below:

$$\text{Emulsion capacity}=\frac{\text{Height of emulsified layer}}{\text{Height of the contents of the tube}}\times 100.$$

The emulsion stability (ES) was measured by recentrifugation as above and heating for 30 min at 80°C. The ES was calculated as shown below:

$$\text{Emulsion stability}=\frac{\text{Height of remaining emulsion layer}}{\text{Height of initial emulsion layer}}\times 100$$

### 2.6 Statistical analysis

All the analyses were replicated at least three times. Statistical analysis was performed with SPSS (Software version 20) using one‐way ANOVA. Duncan multiple‐range test was carried out to compare the mean values for samples with significant differences taken at *p* < .05.

## 3 RESULTS AND DISCUSSION

### 3.1 Proximate composition of defatted conophor nut flours and protein isolates

The protein content of the defatted conophor nut flours ranged from 58.86 to 59.02 g/100 g (Table 1). There was no significant difference in the crude protein content of the defatted conophor nut flours irrespective of the processing techniques. The protein content of the isolates ranged from 86.86 to 87.74 g/100 g, about 1.5‐fold increase when compared to the defatted flour (Table 1). Similar increases in the protein contents of various isolates have been reported with various methods of extraction such as alkali solubilization, isoelectric‐ and micellization‐ precipitation (Wang, Tang, Yang, & Gao, 2008; Teh, Bekhit, Carne, & Birch, 2014; Malomo, He, & Aluko, 2014). The values recorded for the protein content of the isolates in this study is higher than the 80% reported by Gbadamosi et al. (2012b). This may be due to the methods of extraction and crude protein determination. The values, however, compared well with protein isolate from peanut that has been heat processed (Kain, Chen, Sonda, & Abu‐Kpawoh, 2009) and Indian black gram that was subjected to alkali solubilization and isoelectric precipitation (Wani, Sogi, & Gill, 2015). The major determinants of the level of protein in isolates obtainable from legumes are the nature of the plant material and the method of extraction (Malomo & Aluko, 2015). The total ash, crude fiber, and crude fat of the defatted flours were significantly higher than those of the isolates irrespective of the processing technique (Table 1). All the data are in agreement with previous reports on the composition of the isolates of mungbean, lentil, chickpea, pea, and winged bean (Li, Shu, Yan, & Shen, 2010; Karaca, Low, & Nickerson, 2011).

**Table 1:** Proximate composition of heat processed defatted conophor nut flours and protein isolates (g/100 g dry weight basis)

Sample condition	Crude protein	Crude fat	Crude fiber	Total ash	Carbohydrate
*Defatted conophor nut flour*					
Raw	58.95 ± 0.33^b^	3.98 ± 0.12^a^	5.09 ± 0.12^a^	5.04 ± 0.02^b^	26.94 ± 0.16^a^
Boiled	58.86 ± 0.24^b^	3.87 ± 0.02^a^	4.48 ± 0.14^b^	5.55 ± 0.16^a^	27.24 ± 0.24^a^
Toasted	59.02 ± 0.12^b^	3.76 ± 0.06^a^	3.79 ± 0.14^c^	5.78 ± 0.12^a^	27.65 ± 0.26^a^
*Conophor nut protein isolate*					
Raw	86.86 ± 0.48^a^	3.45 ± 0.04^c^	0.02 ± 0.00^d^	3.64 ± 0.03^e^	6.02 ± 0.03^b^
Boiled	86.92 ± 0.36^a^	3.38 ± 0.02^c^	0.01 ± 0.00^d^	3.86 ± 0.01^d^	5.81 ± 0.02^b^
Toasted	87.74 ± 0.28^a^	3.62 ± 0.03^b^	0.02 ± 0.00^d^	3.94 ± 0.02^c^	4.64 ± 0.02^c^

Values are mean ± *SEM* (*n* = 3). Values with different alphabets within a column are significantly different (*p* < .05).

### 3.2 Amino‐acid profile, predicted BV and PER of conophor nut protein isolates

The effect of processing on the amino acid composition of the protein isolates is shown in Table 2. The concentration of valine, leucine, and threonine were particularly higher than other essential amino acids and comparable with the reference protein. Aspartic acid, glutamic acid, and arginine were the highest among the non‐essential amino acids. Similar observation on other plant proteins have been earlier reported (Fagbemi, 2009; Ajibola, Malomo, Fagbemi, & Aluko, 2016). There were increases of up to 18.85%, 33.5%, and 37.5% in the glycine, tryptophan, and histidine, respectively, for isolates of the processed samples. This may be due to chemical reactions as a result of the processing method employed. There were reductions in the other amino acids of conophor proteins ranging from 0.3% to 15.84% in the toasted seed protein with lysine reducing by 15.84% due to heat processing in the toasted sample. High temperature treatments have been reported to reduce lysine and its bioavailability due to formation of Maillard reaction in hazelnut (Özdemir et al., 2001). Fagbemi (2009) has also reported decreases in most of the amino acids of cashewnut when processed by drying, fermentation, and germination.

**Table 2:** Effects of heat processing on the amino acid composition of conophor nut protein isolates (g/100 g)

Amino acids	Raw	Boiled^a^	Toasted^a^	Reference^b^
*Essential amino acid*				
Valine	5.66 ± 0.03	5.27 ± 0.04 (<6.89)	5.57 ± 0.06 (<1.59)	7.6
Leucine	7.76 ± 0.12	7.18 ± 0.08 (<7.47)	7.65 ± 0.14 (<1.42)	8.3
Isoleucine	4.05 ± 0.04	3.43 ± 0.03 (<15.31)	3.95 ± 0.05 (<2.47)	5.6
Phenylalanine	3.64 ± 0.02	4.05 ± 0.05 (>11.26)	3.03 ± 0.02 (<16.76)	5.1
Tryptophan	1.97 ± 0.02	2.57 ± 0.04 (>30.46)	2.63 ± 0.04 (>33.50)	1.8
Methionine	1.27 ± 0.02	1.16 ± 0.04 (<8.66)	1.16 ± 0.02 (<8.66)	3.2
Lysine	3.85 ± 0.03	3.75 ± 0.07 (<2.60)	3.24 ± 0.06 (<15.84)	6.3
Histidine	2.24 ± 0.04	3.08 ± 0.06 (>37.50)	3.04 ± 0.08 (>35.71)	2.4
Threonine	4.68 ± 0.08	4.20 ± 0.03 (<10.26)	4.64 ± 0.06 (<0.85)	5.1
Tyrosine	4.25 ± 0.10	3.87 ± 0.01 (<8.94)	4.37 ± 0.02 (>2.82)	4.0
Cystein	2.18 ± 0.05	2.01 ± 0.01 (<7.80)	1.72 ± 0.03 (<21.10)	1.8
Total essential amino acids	41.55	40.57 (<2.35)	41.00 (<1.32)	51.2
*Non‐essential amino acids*				
Aspartic acid	11.85 ± 1.14	11.66 ± 1.02 (<1.60)	12.46 ± 1.04 (>5.15)	7.6
Serine	6.61 ± 0.09	6.39 ± 0.06 (<3.32)	6.59 ± 0.08 (<0.30)	7.5
Glutamic acid	11.94 ± 0.97	12.54 ± 1.07 (>5.03)	11.77 ± 1.22 (<1.42)	13.5
Proline	7.80 ± 0.05	7.52 ± 0.04 (<3.84)	7.77 ± 0.09 (<0.64)	3.6
Glycine	4.19 ± 0.03	4.98 ± 0.07 (>18.85)	4.98 ± 0.09 (>18.85)	3.6
Alanine	4.53 ± 0.05	4.79 ± 0.08 (>5.74)	4.53 ± 0.04 (0)	6.8
Arginine	11.55 ± 1.05	11.54 ± 0.93 (<0.17)	10.90 ± 0.86 (<5.71)	5.6
Total non‐essential amino acids	58.47	59.42	59.00	48.2
Ratio of essential to non‐essential amino acids	0.71	0.68	0.70	1.06
Arg/Lys	3.00	3.08	3.36	

^a^Values are mean ± SEM (n=3). Values in parenthesis show percentage increase or decrease in the amino acid composition upon processing; ^b^egg was used as reference.

The proportion of the essential amino acid ranged from 40.57% to 41.55%; they were higher than the 36% considered for an ideal protein (FAO/WHO, 2007), but slightly lower than the egg reference of 51.2% (Table 2). The values are higher than the 19% obtained for hull free defatted melon “egusi” flour (*Colocynths citrullus* L.) which is a commonly consumed oil‐seed in Sub‐Saharan Africa for its protein content (Akobundu et al., 1982), hence, conophor nut protein can be regarded as good quality protein. The Arg/Lys ratios obtained in this work are higher than the 2.31, 1.40, <1, 0.44 and <3 reported for hemp, soybean, kidney bean, casein, and rice protein isolates, respectively (Tang, Ten, Wang, & Yang, 2006; Yang et al., 2012; Mundi & Aluko, 2012, 2013). High ratio of Arg/Lys in the diet produces beneficial hypocholesterolemic effects in improving the cardiovascular health (Malomo et al., 2014) and also helps in hypertension regulation (Vallabha, Tapal, Sukhdeo, Govindaraju, & Tiku, 2016). Thus, the present results showed that the conophor nut protein isolates might have positive effects on the cardiovascular system.

The amino acid score of the conophor nut protein isolates from defatted raw, cooked, and toasted flour are presented in Table 3. The isolates are very rich in all the essential amino acids. The scores for cysteine, tyrosine, and tryptophan contents of conophor nut protein isolates were particularly higher than the other amino acids. Processing by boiling or toasting however resulted in a decrease in the sulfur‐containing amino acid (methionine and cysteine) residues in the protein isolates (Table 3). Earlier studies have also reported reduction in methionine and cysteine composition of leguminous seeds after heat processing (Chau, Cheung, & Wong, 1997; Mubarak, 2005). The chemical scores showed that the first limiting amino acids in both the boiled and toasted nuts was methionine (36.25%) while the second limiting amino acid was lysine with 59.52% and 51.43% for the boiled and toasted samples, respectively, when compared to the egg protein reference. Methionine has been reported as a limiting amino acid in the Chinese leguminous plant Dolichos lablab after processing by cooking (Chau et al., 1997).

**Table 3:** Effects of heat processing on the amino acid score, protein efficiency ratio, and biological values of conophor nut protein isolates

Parameters	Raw	Boiled	Toasted	Ref^a^
Amino acids				
Threonine	91.76	82.35	90.98	5.1
Valine	74.47	69.34	73.30	7.6
Methionine	39.69	36.25	36.25	3.2
Lysine	61.11	59.52	51.43	6.3
Cysteine	121.11	111.67	95.56	1.8
Phenylalanine	71.37	79.41	59.41	5.1
Tyrosine	106.25	96.75	109.25	4.0
Isoleucine	72.32	61.25	70.54	5.6
Leucine	93.49	86.51	92.17	8.3
Tryptophan	109.44	142.78	146.11	1.8
First limiting amino acid	Met	Met	Met	
Second limiting amino acid	Lys	Lys	Lys	
Predicted protein efficiency ratio	2.66	2.39	2.55	
Predicted biological value (%)	57.83	73.15	76.97	

^a^Egg was used as reference (FAO/WHO/UNU, 1985).

The predicted BV for conophor nut isolates 57.83%, 73.15%, and 76.97% for the raw, boiled, and toasted samples, and they compared favorably with those reported for common legumes especially soybean curd (Pedroche et al., 2004). The increase in BV of the samples due to boiling and toasting is an indication of improved digestibility potential and effective utilization of the processed nuts over the raw seeds. The predicted protein efficiency ratio ranged from 2.39 to 2.60 and are higher than 2.22–2.34 reported for lupin protein isolates (El‐Adawy, Rahma, El‐Bedawey, & Gafar, 2001), but compares well with the values reported for beach pea and conophor nut protein isolates (Chavan et al., 2001; Gbadamosi et al., 2012b). Hence, conophor nut protein may be a good dietary vegetable protein supplement.

### 3.3 Water and oil absorption capacity and LGC of conophor nuts and protein isolates

In defatted conophor nuts, WAC was lowest (1.18 g/g) and highest (1.36 g/g) in the raw and toasted flours, respectively (Table 4). The increase in the WAC of the toasted conophor nut flour may be due to partial denaturation of proteins during the toasting process which could result in improvement of WAC. During toasting, major proteins can dissociate into subunits with more water binding sites than the native oligomeric proteins; combined with gelatinization of carbohydrates which leads to swelling thus resulting in overall increase in WAC (Akubor, Isolokwu, Ugbane, & Onimawo, 2000). The values obtained for the WAC of the raw, boiled and toasted conophor nut protein isolates are 8.96, 7.34, and 7.54 g/g, respectively (Table 4). These values were significantly (*p* < .05) higher than those of the defatted conophor nut flours. This may be due to the ability of the protein isolates to dissociate and unfold to expose the additional binding sites. The value obtained in this study for WAC of conophor nut protein isolates are higher than 4.2 g/g from *Vicia faba* (Vioque, Alaiz, & Giron‐Calle, 2012), 3.37 g/g from hemp and 4.36 g/g from soybean (Tang et al., 2006), 3.50 g/g from *Phaseolus lunatus* and 2.50 g/g from *Canavalia ensiformis* (Chel‐Guerrero, Perez‐Flores, Betancur‐Ancona, & Davila‐Ortiz, 2002) and 5.5–6.7 g/g from Bambara groundnut (Adebowale, Schwarzenbolz, & Henle, 2011). Thus, the conophor nut protein isolates may find use as functional ingredient in soups, gravies and baked products.

**Table 4:** Functional properties of processed defatted conophor nut and protein isolates

Properties	Defatted conophor nut			Conophor nut protein isolates		
	Raw	Boiled	Toasted	Raw	Boiled	Toasted
Water absorption capacity (g/g)	1.18 ± 0.02^d^	1.25 ± 0.07^c^	1.36 ± 0.08^c^	8.96 ± 0.35^a^	7.34 ± 0.34^b^	7.54 ± 0.24^b^
Oil absorption capacity (g/g)	1.00 ± 0.06^b^	1.07 ± 0.02^b^	1.08 ± 0.06^b^	6.11 ± 0.11^a^	6.21 ± 0.02^a^	6.24 ± 0.16^a^
Foaming capacity (%)	11.54 ± 0.39^c^	13.06 ± 0.61^b^	14.91 ± 0.66^a^	4.21 ± 0.02^f^	4.40 ± 0.01^e^	4.65 ± 0.01^d^
Foaming stability (%)	5.70 ± 0.16^a^	4.67 ± 0.12^b^	3.98 ± 0.06^c^	2.87 ± 0.01^d^	2.49 ± 0.01^e^	2.11 ± 0.01^f^
Emulsion capacity (%)	9.65 ± 0.06^b^	10.44 ± 0.07^a^	10.53 ± 0.05^a^	4.86 ± 0.07^c^	4.68 ± 0.01^cd^	4.52 ± 0.02^d^
Emulsion stability (%)	4.11 ± 0.03^b^	3.89 ± 0.10^c^	2.59 ± 0.01^e^	4.65 ± 0.02^a^	4.63 ± 0.1^a^	3.52 ± 0.01^d^
Least gelation capacity (%)	18.50 ± 0.72^a^	16.68 ± 0.06^b^	12.82 ± 0.13^d^	18.15 + 0.02^a^	16.28 ± 0.08^b^	14.90 ± 0.03^c^
Bulk density (g/ml)	0.36 ± 0.02^e^	0.44 ± 0.01^d^	0.58 ± 0.02^c^	0.62 ± 0.01^c^	0.65 ± 0.01^a^	0.67 ± 0.02^a^

Values are mean ± *SEM* (*n* = 3). Values with different alphabets on a row are significantly different (*p* < .05).

The OAC of the raw, boiled, and toasted conophor nut protein isolates are 6.11, 6.21, 6.24 g/g, respectively while the defatted conophor nut flours had OAC of 1.00, 1.07, 1.08 g/g, respectively (Table 4). The higher protein contents of the isolates compared to those of defatted flours (Table 1) could have aided the protein‐protein interactions that contributed to the high OAC of the isolates. It may also be due to the exposure of more hydrophobic amino acids. The values obtained for OAC of the defatted flour samples in this study were lower than 2.00 g/g reported for defatted niger seed flour (Bhagya & Sastry, 2003). The OAC values obtained for conophor nut protein isolates are higher than 5.2 g/g from hemp and 5.32 g/g from soybean (Tang et al., 2006), 2.2–7.2 g/g for Bambara groundnut (Adebowale et al., 2011), 1.12 g/g from mungbean (Akaerue & Onwuka, 2010) and 4.6 g/g from *Canavalia ensiformis* (Chel‐Guerrero et al., 2002) protein isolates. The values, however, compare well with the 5.5–6.3 g/g reported for Indian black gram protein isolate (Wani et al., 2015). Although, the values obtained were lower than 13.7 g/g reported for hemp protein isolate (Malomo et al., 2014), the conophor protein isolate is still laden with potential to be applied as ingredient in pie‐filling, meat extenders and meat substitutes for sausages, where the protein could bridge the fat and water contents preventing the loss of both.

### 3.4 Foaming capacity and foam stability

Foam capacity is a measure of the increase observed when 1% of protein isolate is whipped in 100 ml water while foam stability is a measure of the volume of foam remaining, relative to the initial state after the whipping, when the solution is left for a specified period of time. A protein with good foaming properties and flexible surfactant molecules must be rapidly adsorbed at the air‐water interface and rearranged to form a cohesive visco‐elastic film via intermolecular interactions (Malomo et al., 2014). The FC of the defatted flours ranges from 11.54–14.91%, and was improved by boiling and toasting. The FC of defatted conophor nuts flour compared favorably with 13.2% reported for fluted pumpkin flours (Oshodi & Fagbemi, 1992). The corresponding protein isolates had poor FC of 4.21–4.65% (Table 4), albeit with improvement upon boiling and toasting. Foams are very vital in foods like whipped toppings and beverages where the proteins unfold forming a layer that keep air bubbles in suspension and prevents them from collapsing. The stabilized foams of the conophor protein flours (3.98%–5.70%) and isolates (2.11%–2.87%) were observed to be very low when compared to 95%–100% for hemp protein isolates (Malomo et al., 2014). The poor FS of the flours and isolates is indicative of the very slow film formation at the air‐water interfaces coupled with poor film visco‐elasticity (Wani et al., 2015).

### 3.5 Emulsification properties

The emulsion capacities of the raw, boiled, and toasted defatted nuts were 9.65%, 10.44% and 10.53%, respectively. Toasting significantly decreased the emulsion capacity and stability of the conophor nut protein isolates, but significantly (*p* < .05) increased the emulsion capacity of the defatted flour (Table 4). The emulsion formed by the protein isolate when the nuts were boiled was more stable which might be due to the presence of strong protein–protein interactions at the oil–water interface of the protein isolates preventing oil droplet coalescence (Malomo et al., 2014). The nature of the proteins with the composition of charged, non‐charged polar and non‐polar amino acids make them potential emulsifiers and surfactants possessing both hydrophilic and hydrophobic properties and are able to interact with both water and oil components in food systems (Ulloa, Rosas‐Ulloa, & Ulloa‐Rangel, 2011).

### 3.6 Least gelation concentration and bulk density

Gelation occurs when proteins form a three‐dimensional network that is resistant to flow under pressure and least gelation concentration (LGC) is used as an indication of the gelation capacity of food protein (Boye et al., 2010). The LGC of defatted raw, boiled, and toasted conophor flours are 18.50%, 16.68%, and 12.82%, respectively, while for the protein isolates they were 18.15%, 16.28% and 14.90% for the raw, boiled, and toasted samples, respectively. There was no significant difference between the LGC of the raw and boiled samples of the defatted flour and protein isolates. The toasted samples had the lowest LGC for the defatted flour and the protein isolate. The present values showed conophor protein isolates as very poor gelling agent compared to 10% for soybean flour and safflower (Padilla, Alvarez, & Alfaro, 1996; Ulloa et al., 2011) and 13.5% for cashewnut protein isolates (Ogunwolu, Henshaw, Mock, Santros, & Awonorin, 2009), but better gelling than the 22% for hemp protein isolate (Malomo et al., 2014) because, the lower the LGC the better the gelling ability. The gelation properties are dependent on the nature of the protein and other non‐protein components of the sample (Omowaye‐Taiwo et al., 2015).

The bulk density (BD) of the defatted flours ranged from 0.36% to 0.58 g/ml (Table 4) with the lowest and highest BD in raw and toasted conophor nut flours, respectively. These values are higher than earlier report for ginger bread plum and peanut meal (Amza, Amadou, Kamara, Zhu, & Zhou, 2011). The BD was however improved in the protein isolates with values of 0.62, 0.65 and 0.67 g/ml for the raw, boiled, and toasted samples, respectively. These values are higher than 0.31 g/ml reported for cashew nut (Ogunwolu et al., 2009) and 0.27 g/ml for safflower (Ulloa et al., 2011). The BD is an indication of the porosity of a product that influences package design and could be used in determining the type of packaging material required for the product. Higher bulk densities are usually desirable in the food industries for the packaging advantages they offer (Fagbemi, 1999).

## 4 CONCLUSION

The present investigation has revealed that conophor nut has the potential for the preparation of plant protein isolates with protein content of over 80%. The isolates have good water and oil absorption, and foaming capacities that were higher than those of other oil seeds. Boiling and toasting only improved the foam stability of the defatted flour but not the protein isolates. The tyrosine and tryptophan contents of the protein isolates were increased as a result of heat processing. Protein isolates from raw, boiled and toasted conophor nut contain substantial amount of essential amino acids required in food for healthy nutrition. The isolates also exhibited higher level of biological value and protein efficiency ratios. Thus, the improved nutritional and functional properties of the conophor nut protein isolates by heat processing may enhance its suitability as novel functional ingredients for the food system at local level and for industrial applications.

## CONFLICT OF INTEREST

None declared.
